# Endogenous generation of hydrogen sulfide and its regulation in *Shewanella oneidensis*

**DOI:** 10.3389/fmicb.2015.00374

**Published:** 2015-04-28

**Authors:** Genfu Wu, Ning Li, Yinting Mao, Guangqi Zhou, Haichun Gao

**Affiliations:** Institute of Microbiology and College of Life Sciences, Zhejiang UniversityHangzhou, China

**Keywords:** H_2_S, endogenous generation, regulation, Crp, *Shewanella*

## Abstract

Hydrogen sulfide (H_2_S) has been recognized as a physiological mediator with a variety of functions across all domains of life. In this study, mechanisms of endogenous H_2_S generation in *Shewanella oneidensis* were investigated. As a research model with highly diverse anaerobic respiratory pathways, the microorganism is able to produce H_2_S by respiring on a variety of sulfur-containing compounds with SirACD and PsrABC enzymatic complexes, as well as through cysteine degradation with three enzymes, MdeA, SO_1095, and SseA. We showed that the SirACD and PsrABC complexes, which are predominantly, if not exclusively, responsible for H_2_S generation via respiration of sulfur species, do not interplay with each other. Strikingly, a screen for regulators controlling endogenous H_2_S generation by transposon mutagenesis identified global regulator Crp to be essential to all H_2_S-generating processes. In contrast, Fnr and Arc, two other global regulators that have a role in respiration, are dispensable in regulating H_2_S generation via respiration of sulfur species. Interestingly, Arc is involved in the H_2_S generation through cysteine degradation by repressing expression of the *mdeA* gene. We further showed that expression of the *sirA* and *psrABC* operons is subjected to direct regulation of Crp, but the mechanisms underlying the requirement of Crp for H_2_S generation through cysteine degradation remain elusive.

## Introduction

Hydrogen sulfide (H_2_S), traditionally recognized as a noxious gas, is now regarded as an important signaling molecule along with nitric oxide (NO) and carbon monoxide (CO), associated with beneficial functions in mammals from vasorelaxation, cardioprotection, and neurotransmission to anti-inflammation (Wang, 2012). In bacteria, investigations into its physiological roles just began. It has been recently elucidated that H_2_S confers bacterial cells defense against antibiotics by stimulating the cellular protection system against reactive oxygen species (ROS) (Shatalin et al., 2011). Beyond this, our understanding of the mechanism by which H_2_S affects biology is limited. In both eukaryotes and prokaryotes, H_2_S can be produced endogenously via assimilatory sulfate reduction and cysteine degradation. H_2_S of a very small amount formed via the former is rapidly assimilated into organic sulfur compounds such as sulfur-containing amino acids and is hardly released into extracellular environments. On the contrary, the latter, catalyzed by cystathionine β-synthase (CBS), cystathionine γ-lyase (CSE), and 3-mercaptopyruvate sulfurtransferase (3MST) together with cysteine aminotransferase (CAT), is responsible for H_2_S generation on a large scale, ensuring that the molecule can be functionally implicated in many physiological processes (Shatalin et al., 2011; Kimura, 2014). Most, if not all, of bacterial genomes encode some of these enzymes. For example, *Bacillus anthracis*, *Pseudomonas aeruginosa*, and *Staphylococcus aureus* have the CBS/CSE operon but not 3MST, whereas *Escherichia coli* carries 3MST, but not CBS/CSE (Shatalin et al., 2011).

Some prokaryotes have an additional route for H_2_S generation on a large scale, the dissimilatory sulfate/sulfite (SO^2−^_4_/SO^2−^_3_) reduction, which is best illustrated in sulfate-reducing bacteria (SRB) *Desulfovibrio vulgaris* (Bradley et al., 2011). The dissimilatory sulfite reductases, featuring siroheme and [4Fe-4S] prosthetic centers, are minimally composed of two subunits (DsrA and DsrB) in an α_2_β_2_ arrangement and catalyze the six-electron reduction of sulfite to sulfide (Oliveira et al., 2008). In addition, two other enzyme complexes, represented by PsrABC of *Salmonella enterica* Serovar Typhimurium and SirACD of *Shewanella oneidensis*, have been identified to be able to reduce various inorganic sulfur species to H_2_S (Heinzinger et al., 1995; Shirodkar et al., 2011). The former is able to use as electron acceptors (EAs) thiosulfate (S_2_O^2−^_3_), tetrathionate (S_4_O^2−^_6_), and elemental sulfur (S^0^) for respiration while the latter, which shares similarities with the formate-dependent nitrite reductase, catalyzes direct conversion of sulfite to sulfide. PsrC and SirD serve as quinol oxidases that transfer electrons stepwise via PsrB and SirC to the catalytic subunit PsrA and SirA for reduction of corresponding EAs, respectively (Jormakka et al., 2008; Cordova et al., 2011).

*S.oneidensis*, a facultative Gram-negative γ-proteobacterium, has been intensively studied owing to its exceptional metabolic flexibility and its potential use for the bioremediation of metal/radionuclide contaminants in the environment (Fredrickson et al., 2008). Although the microorganism neither belongs to SRB phylogenetically nor possesses analogs of DsrAB, under anaerobic conditions it generates H_2_S from various sulfur compounds including thiosulfate, sulfite, tetrathionate, and elemental sulfur, a feature firstly revealed more than two and half decades ago (Myers and Nealson, 1988). However, the enzymatic foundation for the reduction was not revealed until recently (Burns and DiChristina, 2009; Shirodkar et al., 2011). In addition to SirACD mentioned above, *S.oneidensis* also relies on a thiosulfate and polysulfide reductase (PsrABC), homologous to that from *S. enterica* Serovar Typhimurium (Heinzinger et al., 1995). Based on available genome sequences, it is clear that most of *Shewanella* species, if not all, are able to produce H_2_S endogenously via respiration of sulfur species (Gao et al., 2010a).

Most recently, endogenous H_2_S production has been linked to iron reduction in *S. oneidensis* and *Sulfurospirillum deleyianum* (Flynn et al., 2014; Lohmayer et al., 2014). In alkaline environments containing sulfur species and iron compounds, these bacteria first generates H_2_S (HS^−^), which subsequently reduces iron compounds abiotically (Flynn et al., 2014), indicating an essential role of H_2_S in bacterial metal reduction under certain conditions. In contrast to the beneficial role, Shewanellae are increasingly being implicated as human pathogens in individuals exposed through occupational or recreational activities to marine niches where these species thrive (Janda and Abbott, 2014). Additionally, *Shewanella* have been recognized as spoilage bacteria of food (especially marine products) and associated with foul odors, which are in part attributed to endogenous H_2_S (Janda and Abbott, 2014).

Hence, understanding endogenous H_2_S production would not only provide insights into biogeochemical redox processes related to metal reduction, but also help to deter and mitigate the emerging threat to human health. In this study, we made efforts to provide a relatively comprehensive understanding of endogenous H_2_S production and regulation in *S.oneidensis*. We found that the bacterium possesses a large set of enzymes catalyzing H_2_S generation through cysteine degradation in addition to anaerobic respiration of sulfur-containing compounds. While respiration predominantly, if not exclusively, relies on the SirACD and PsrABC complexes there is no interplay with each other. We showed that global regulator Crp (cyclic-AMP receptor protein) but not Fnr (fumarate nitrate regulator) or Arc (aerobic respiration control) is essential to H_2_S generation via anaerobic respiration. Additionally, we found that both Crp and Arc are involved in H_2_S generation through cysteine degradation. While it is clear that Arc exerts its impact on the process by repressing expression of the major contributor, the mechanisms underlying the requirement of Crp remain unknown.

## Methods and materials

### Bacterial strains, plasmids, and culture conditions

A list of all bacterial strains and plasmids used in this study is given in Table 1. All chemicals were acquired from Sigma Co. (Shanghai, China) unless otherwise noted. Information for primers used in this study is available upon request. For genetic manipulation, *E. coli* and *S. oneidensis* strains under aerobic conditions were grown in Lysogeny Broth (LB, Difco, Detroit, MI) medium (Bertani, 2004), which was modified to contain tryptone (10 g/L), yeast extract (5 g/L), and NaCl (5 g/L), at 37 and 30°C, respectively. When needed, the growth medium was supplemented with chemicals at the following concentrations: 2,6-diaminopimelic acid (DAP), 0.3 mM; ampicillin sodium, 50 μg/ml; kanamycin sulfate, 50 μg/ml; and gentamycin sulfate; 15 μg/ml.

**Table 1:** **Strains and plasmids used in this study**.

**Strain or plasmid**	**Description**	**Reference or source**
***E. coli* STRAINS**		
DH5α	Host strain for plasmids	Lab stock
WM3064	Donor strain for conjugation; Δ*dapA*	W. Metcalf, UIUC
XL1-Blue MRF'Kan	Recipient strain for two-hybrid system	Stratagene
***S. oneidensis* STRAINS**		
MR-1	Wild-type	ATCC 700550
HG0479	Δ*sirA* derived from MR-1	This study
HG0624	Δ*crp* derived from MR-1	Gao et al., 2010b
HG1095	ΔSO_1095 derived from MR-1	This study
HG1261	Δ*sseA* derived from MR-1	This study
HG1329	Δ*cyaC* derived from MR-1	This study
HG1812	Δ*mdeA* derived from MR-1	This study
HG2191	Δ*metC* derived from MR-1	This study
HG2356	Δ*fnr* derived from MR-1	Gao et al., 2010b
HG2903	Δ*cysK* derived from MR-1	This study
HG3598	Δ*cysM* derived from MR-1	This study
HG3988	Δ*arcA* derived from MR-1	Gao et al., 2008
HG4056	Δ*metB* derived from MR-1	This study
HG4062	Δ*psrA* derived from MR-1	This study
HG1095-1812	Δ*mdeA*ΔSO_1095 derived from MR-1	This study
HG1261-1812	Δ*mdeA*Δ*sseA* derived from MR-1	This study
HG0479-4062	Δ*sirA*Δ*psrA* derived from MR-1	This study
HG1329-4312	Δ*cyaC*Δ*cyaA* derived from MR-1	This study
**PLASMIDS**		
pHGM01	Ap^R^, Gm^R^, CM^R^, suicide vector	Jin et al., 2013
pHG101	Promoterless broad-host Km^R^ vector	Wu et al., 2011
pHG102	pHG101 containing the *S. oneidensis arcA* promoter	Wu et al., 2011
pHGT01	Promoter-embedded Mariner-based transposon vector	Yin et al., 2015
pBXcmT	B1H bait vector	Guo et al., 2009
pTRG	B1H target vector	Stratagene
pBXcmT-P*_cyd_*	Positive control bait vector	Jiang et al., 2014
pBXcmT-P*_16s_*	Negative control bait vector	Li et al., 2014
pTRG-Crp	B1H target vector expressing *crp*	Jiang et al., 2014
pHGEI01	Integrative *lacZ* reporter vector	Fu et al., 2014
pBBR-Cre	Helper vector for antibiotic marker removal	Fu et al., 2013

### Physiological characterization of *S. oneidensis* strains

M1 defined medium containing 0.02%(w/v) of vitamin free casamino acids and 20 mM lactate was used as described previously (Gao et al., 2008). Growth of *S. oneidensis* strains under aerobic or anaerobic conditions was determined by recording optical densities of 600 nm (OD_600_) of cultures. For aerobic growth, fresh media were inoculated to ~0.01 of OD_600_ with overnight cultures grown from a single colony. For anaerobic growth, exponential phase cultures grown aerobically were centrifuged, washed with fresh medium twice, purged in nitrogen and suspended in fresh medium to ~0.01 of OD_600_ in an anaerobic glove box. To cultivate Δ*crp* and Δ*cyaC* under anaerobic conditions, Trimethylamine N-oxide (TMAO) was used as growth supporting EA. EAs were used at the concentration of 10 mM unless otherwise noted.

### Mutagenesis and genetic complementation

*S. oneidensis* in-frame deletion strains were constructed using the *att*-based Fusion PCR method (Jin et al., 2013). In brief, two fragments flanking the target gene were generated by PCR with primers containing *attB* and the gene specific sequence, and then were joined by a second round of PCR. The fusion fragments were introduced into plasmid pHGM01 by site-specific recombination using the BP Clonase (Invitrogen) according to the manufacturer's instruction. The resulting mutagenesis vectors were transformed into *E. coli* WM3064 and the verified ones were transferred into proper *S. oneidensis* strains via conjugation. Integration of the mutagenesis constructs into the chromosome were selected by resistance to gentamycin and confirmed by PCR. Verified transconjugants were grown in LB broth in the absence of NaCl and plated on LB supplemented with 10% sucrose. Gentamycin-sensitive and sucrose-resistant colonies were screened by PCR for the intended deletion. All mutations were verified by sequencing the mutated regions.

Mutants from previous studies were successfully complemented by using either multiple-copy or single-copy (integrative) vectors (Table 1). In this study, the same complementation constructs were used and similar results were obtained as indicated in the figure legends. For newly constructed mutants, plasmids pHG101 and pHG102 were utilized for genetic complementation (Wu et al., 2011). For genes adjacent to their promoter, a fragment containing the gene of interest and its native promoter was generated by PCR and cloned into pHG101. For others, the gene of interest was amplified and inserted into the multiple-cloning site (MCS) of pHG102 under the control of the *arcA* promoter, which is constitutively active (Gao et al., 2010b). After verified by sequencing, the resulting complementation vector was transferred into its relevant mutant strains via conjugation.

### H_2_S detection

H_2_S generation in *S. oneidensis* strains was monitored by using a lead acetate detection method (Shatalin et al., 2011). Paper strips saturated by 2% of Pb(Ac)_2_ were affixed to the inner wall of a cultural tube, above the level of the liquid culture. Overnight cultures were used to inoculate fresh LB media to ~0.01 of OD_600_ and incubated for ~20 h at 30°C with aeration. Stained paper strips were scanned and quantified with a UVP HR410 Imaging System (UVP). The results were normalized to OD_600_ readings. H_2_S concentrations in liquid cultures grown under aerobic and anaerobic conditions were quantified by using the methylene blue formation assay (Siegel, 1965). In brief, properly diluted aliquots (1.6 ml) were mixed with 0.2 ml DPD (20 mM *N*, *N*-dimetyl-*p*-phenylenediamine sulfate in 7.2 M HCl) and 0.2 ml FeCl_3_ (30 mM FeCl_3_ in 1.2 M HCl). After 30 min at 25°C, the absorbance at 667 nm was measured and related to sulfide concentration using calibration curves generated with NaHS.

### Transposon mutagenesis

A random transposon-insertion library was constructed with pHGT01, a mariner-based transposon vector which carries an embedded promoter within the transposable fragment (Fu et al., 2013; Yin et al., 2015). When mutants were grown in LB containing SO^2−^_3_, S_2_O^2−^_3_, and cysteine at 2 mM to the late exponential phase in 96-well plates, the lids of the plates were removed to let out gaseous H_2_S, and 1 h later the H_2_S levels of cultures in each well were visualized with paper strips saturated by 2% of Pb(Ac)_2_. Mutants exhibiting significant reduction in H_2_S generation were saved and then cultured in individual tubes for verification. The confirmed mutants were subjected to arbitrary PCR for mapping the transposon insertion sites (Das et al., 2005).

### B1H assay

Bacterial one-hydrid system was used to investigate DNA-protein interaction *in vivo* in *E. coli* cells (Guo et al., 2009). Briefly, plasmid constructs were created by cloning the bait DNA and target Crp into the pBXcmT and pTRG vectors, respectively, and verified by sequencing. The resultant plasmids were used to co-transform BacterioMatch II Validation Reporter Competent Cells on M9 salt agar plates containing 25 mg/ml chloramphenicol and 12.5 mg/ml tetracycline with or without 3-AT. A pair of pBXcmT-P*_cyd_*/pTRG-Crp were used as positive control, and a 300 bp DNA fragment of the 16s rRNA gene promoter (pBXcmT-P_16*s*_) was used as negative control (Jiang et al., 2014; Li et al., 2014). The plates were incubated for 24 h and then moved to room temperature for an additional 16 h (colonies indicating positive interaction usually appeared between 18 and 24 h). The positive interactions were confirmed by streaking colonies on plates containing both 3-AT and streptomycin (12.5 mg/ml).

### Expression analyses

To prepare samples for expression analyses, cultures at the mid-log phase were aliquotted; an aliquot was used as the untreated control and other aliquots were added with chemicals indicated in the text, and then collected 30 min post-addition. In total, four biological replicates under each condition were collected for the analyses. Levels of gene expression were determined by using an integrative *lacZ*-reporter system and β-Galactosidase activity assay, as performed essentially the same as before (Fu et al., 2014; Jiang et al., 2014). In addition, levels of mRNAs were also assayed using Quantitative RT-PCR (qRT-PCR) with an ABI7300 96-well qRT-PCR system (Applied Biosystems) as described previously (Yuan et al., 2011).

### Other analyses

DNA and protein sequence similarity searches were performed using the BLAST program. Sequences of proteins of interest for alignments were obtained from Genbank. Alignments were performed using Clustal Omega (http://www.ebi.ac.uk/Tools/msa/clustalo). Promoter prediction for genes of interest was performed by using the Neural Network Promoter Prediction program (Reese, 2001). Experimental values are subjected to statistical analyses and presented as means ± SD (standard deviation). Student's *t*-test was performed for pairwise comparisons of groups.

## Results

### MdeA is the major contributor for H_2_S generation under aerobic conditions

**Figure 1:**
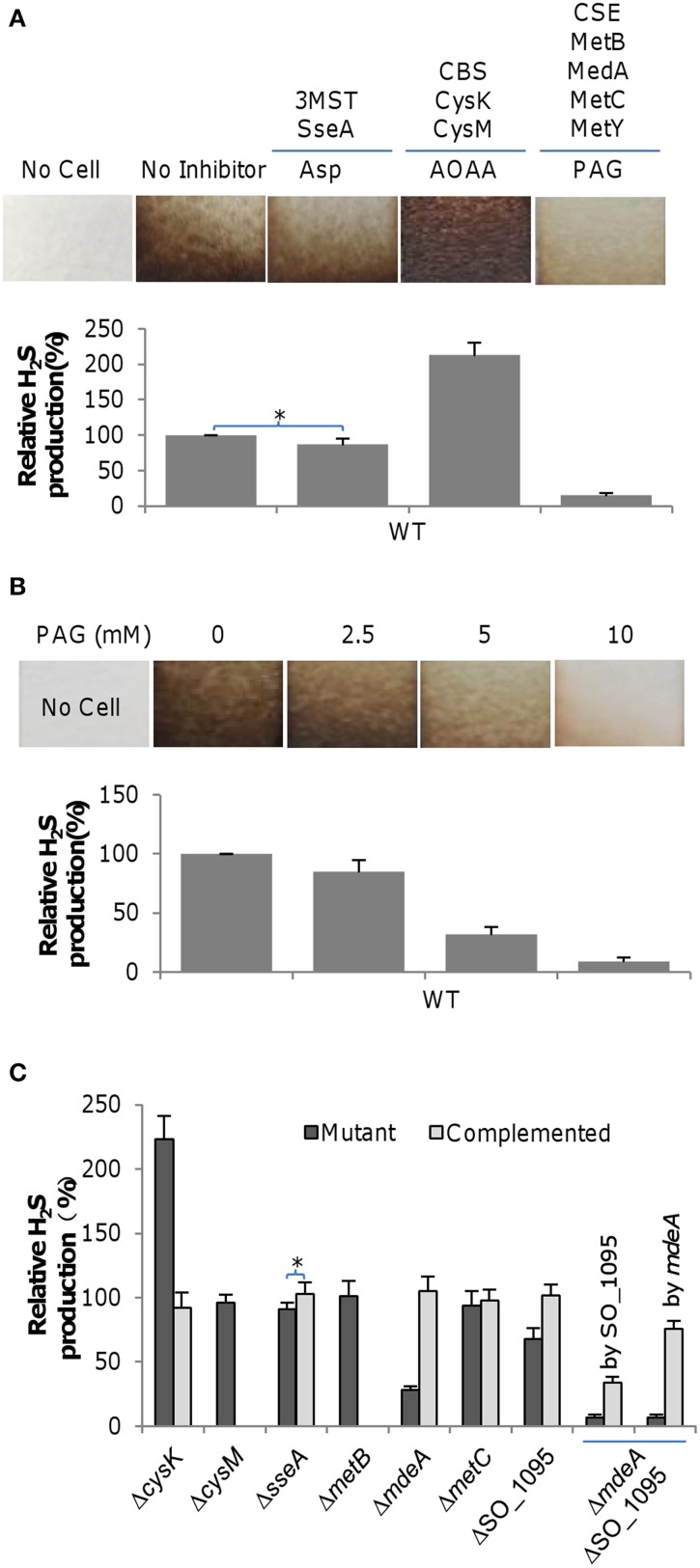
**H_2_S generation by *S. oneidensis*. (A)** Effects of Asp (3 mM), AOAA (50 μM), and PAG (7.5 mM), inhibitors of 3MST, CBS, and CSE respectively, on H_2_S generation by the *S. oneidensis* wild-type. **(B)** Effects of PAG, the CSE inhibitor, at varying concentrations on H_2_S generation by the *S. oneidensis* wild-type. **(C)** H_2_S generation by indicated *S. oneidensis* mutants. Mutants showing a significant defect in H_2_S generation were subjected to genetic complementation. The double mutant (Δ*mdeA*ΔSO_1095) was complemented by either gene separately. Lead acetate–soaked paper strips show a PbS brown or black stain as a result of reaction with H_2_S. Strips were affixed to the inner wall of a culture tube, above the level of the liquid cultures for ~20 h. Levels of the wild-type were averaged and set to be 100%, and used to normalize averaged H_2_S levels of all other samples. In **(C)**, the methylene blue formation assay was used for quantification of H_2_S. Data are presented as the mean ± SD from at least five independent experiments. Asterisks indicate statistically significant difference (*, *p* < 0.05).

Given that the assimilatory reduction of sulfite usually does not release H_2_S into the environment, endogenous H_2_S at physiologically relevant levels in cultures is predominantly produced by reductases reducing sulfur-containing compounds and orthologs of mammalian H_2_S generation enzymes (cysteine degradation), including CBS, CSE, and 3MST (Shatalin et al., 2011; Keseler et al., 2013) (Figure S1). In *S. oneidensis*, thiosulfate and polysulfite reductase PsrABC and sulfite reductase SirACD are known to be responsible for H_2_S generation under anaerobic conditions (Burns and DiChristina, 2009; Shirodkar et al., 2011). In contrast, the pathways for H_2_S generation through the cysteine degradation pathway remain veiled. To identify orthologs of mammalian cysteine degradation enzymes, we performed BLASTp screening using *E. coli* 3MST and *P. aeruginosa* CBS and CSE against the *S. oneidensis* proteome. Results showed that the genome encodes a set of high confidence hits for these three proteins (Table S1 and Figure S2). Multiple orthologs of high confidence for CBS (CysM and CysK) and CSE (MetB, MdeA, SO_1095, and MetC) are present while there is only one for 3MST (SseA). This is not surprising given that the *E. coli* genome also encodes several proteins homologous to these three enzymes (Shatalin et al., 2011). However, it is common that only a portion of the homologous proteins are enzymes involved in H_2_S generation, based on the findings from *E. coli*, *B. anthracis*, *P. aeruginosa*, and *S. aureus* (Shatalin et al., 2011). To assess the contribution of the predicted enzymes in H_2_S generation through cysteine degradation under aerobic conditions, we inactivated each set of enzymes chemically using their specific inhibitors: amino-oxyacetate (AOAA) for CBS, asparate (Asp) for 3MST, and DL-propargylglycine (PAG) for CSE (Shatalin et al., 2011). H_2_S generation was detected using lead acetate [Pb(Ac)_2_], which can specifically react with H_2_S to form brown-colored precipitate of lead sulfide [PbS], whose amount is proportional to the concentration of H_2_S. As shown in Figure 1A, effects of these inhibitors on H_2_S generation varied significantly. Compared to the non-inhibitor control culture, addition of 7.5 mM PAG nearly eliminated PbS staining (<10%) and Asp had a rather mild impact on H_2_S generation, manifesting contribution of CSE and 3MST in H_2_S generation. In contrast, addition of AOAA increased H_2_S generation significantly, implicating that the CBS homologs do not function as cystathionine β-synthase. To confirm that CSE is the major source of H_2_S generation, we assessed the effects of PAG of various concentrations. As expected, the amounts of H_2_S produced were inversely proportional to the concentrations of the inhibitor (Figure 1B). In parallel, H_2_S generation was also quantified in liquid static media in 24-well plates by using the methylene blue formation assay (Siegel, 1965). We found that data from these two methods were comparable in general. In the rest of the study, the methylene blue formation assay was chosen because it is more sensitive and easier for quantification.

To decisively determine roles of these enzymes in H_2_S generation under aerobic conditions, we constructed a set of mutants that lack one of these enzymes and monitored H_2_S production in the resulting mutants (Figure 1C). All mutants were indistinguishable from the wild-type with respect to growth under aerobic or anaerobic conditions with fumarate as the sole EA (data not shown). Among all mutants, the Δ*cysK* strain stood out as it produced substantially more H_2_S than the wild-type. This phenotype, similar to that resulting from the addition of AOAA, was confidently attributed to the *cysK* mutation after successful genetic complementation. The result, however, is not surprising because CysK primarily catalyzes the synthesis of L-cysteine from O-acetyl-L-serine and H_2_S although it has also been implicated in H_2_S generation (Byrne et al., 1988) (Figure S1). Consistent with the effect of Asp, a mutational analysis of the *sseA* gene revealed that the protein had a negligible role in H_2_S generation. In the case of CSE analogs, MetB and MetC appeared to be dispensable for H_2_S generation, a scenario agreeing with their annotated functions (Figure S1). In contrast, loss of either MdeA or SO_1095 resulted in drastic reduction in H_2_S generation, indicating that these two proteins are major contributors for releasing endogenous H_2_S. Apparently, MdeA is the most important enzyme for the task in *S. oneidensis*, accountable for at least 70% of H_2_S generation under test conditions. The involvement of MdeA, a methionine γ-lyase, in the process is reasonable because it converts L-cysteine and H_2_O to pyruvate, NH_3_, and H_2_S (Figure S1) (Sato and Nozaki, 2009). However, it is not immediately evident why SO_1095 contributes to H_2_S generation given that its predicted role as O-acetylhomoserine (thiol)-lyase is not related to any known pathway for H_2_S generation. The observed phenotypes of reduced H_2_S generation resulting from the *mdeA* and SO_1095 deletions were corrected by their expression *in trans*, validating the linkage between the phenotypes and corresponding mutations. To further confirm that MdeA and SO_1095 are enzymes predominantly responsible for endogenous H_2_S generation in *S. oneidensis*, we constructed a strain lacking both the *mdeA* and SO_1095 genes. Not surprisingly, the double removal further compromised the capacity of H_2_S generation (Figure 1C). These data, collectively, indicate that in *S. oneidensis MdeA* and SO_1095 dictate endogenous H_2_S generation.

### H_2_S generation pathways with MdeA and SseA are cysteine-inducible

**Figure 2:**
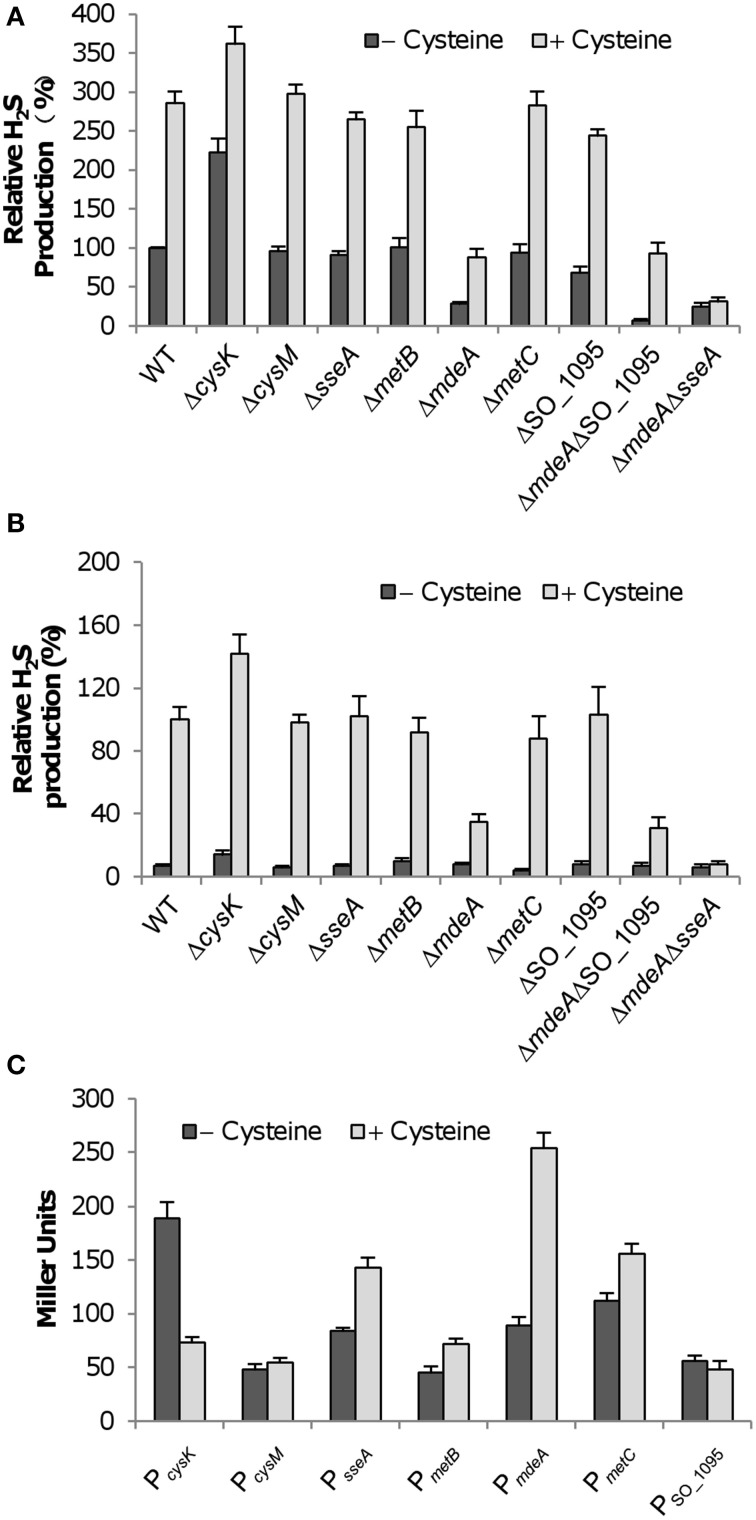
**Effect of cysteine on *S. oneidensis* strains. (A)** Effect of 10 mM cysteine on H_2_S generation by *S. oneidensis* strains under aerobic conditions. Averaged H_2_S level of the wild-type in the absence of cysteine was set to 100% for subsequent normalization. **(B)** Effect of 10 mM cysteine on H_2_S generation by *S. oneidensis* strains under anaerobic conditions. Fumarate was used as the sole EA. Averaged H_2_S level of the wild-type in the presence of cysteine was set to 100% for subsequent normalization because H_2_S levels were extremely low without induction. **(C)** Effect of 10 mM cysteine on activities of indicated promoters. For each test gene, the promoter-*lacZ* (*E. coli*) construct was introduced into the chromosome of the wild-type and the activities of the promoter as single copy were assessed by β-galactosidase assay. Data are presented as the mean ± SD from at least three independent experiments.

The primary source of endogenous H_2_S is L-cysteine; as a consequence, many processes leading to H_2_S generation are subjected to induction by the amino acid (Shatalin et al., 2011). To determine which enzymes are cysteine-inducible, we measured the H_2_S generation capacity of *S. oneidensis* strains with cysteine. In the presence of 10 mM cysteine, there was an increase of ~2.8-fold in H_2_S generation in the wild-type (Figure 2A). Similar results were obtained from mutant strains in which mutations do not show any noticeable impact on H_2_S generation, confirming that these genes are not involved in the process. Additionally, induction to comparable levels by L-cysteine was also observed in the Δ*sseA* and ΔSO_1095, supporting that MdeA is the enzyme responsible for increased H_2_S generation. In both the Δ*mdeA* and Δ*mdeA*ΔSO_1095 strains, the induction decreased to similar levels, implicating that SO_1095 may not be subjected to induction by L-cysteine. Rather, this induction is likely attributed to SseA. To test it, we generated an *mdeA* and *sseA* double mutant (Δ*mdeA*Δ*sseA*) and assayed its capacity of H_2_S generation (Figure 2A). In the absence of cysteine, the mutant generated H_2_S at a level similar to that from the *mdeA* single mutant. Addition of cysteine did not result in any increase in H_2_S generation, indicating that the mutant is reluctant to respond to exogenous cysteine. These data manifest that SseA becomes a significant contributor for H_2_S generation under induced conditions.

H_2_S generation through cysteine degradation is supposed to be independent of EAs for respiration. We therefore reasoned that the induction of H_2_S generation by L-cysteine should be observed under anaerobic conditions as well. To test this, the wild-type and mutant strains were assayed for H_2_S generation with fumarate as the sole EA. As shown in Figure 2B, H_2_S was hardly generated in any of these strains in the absence of L-cysteine, most likely because levels of metabolically synthesized substrates for cysteine degradation enzymes are too low to elicit a significant influence on overall H_2_S generation during growth. When cysteine was supplemented, levels of H_2_S in cultures of all test strains increased drastically (Figure 2B). Compared to the data obtained from the aerobic cultures, a similar trend was observed, confirming that the activities of MdeA and SseA are EA-independent and cysteine-inducible.

To further investigate into the effect of L-cysteine on H_2_S generation in *S. oneidensis*, we employed an integrative *lacZ*-reporter system pHGEI01 to assess expression of these genes (Fu et al., 2014). The reporter system is characterized by its ability to integrate into the chromosome and a removable antibiotic marker, and thus allows us to avoid complications of antibiotics on growth and of copy numbers of plasmids on *lacZ* expression. All of these genes are immediately adjacent to the promoters of their residing operons (data not shown). According to the promoter prediction, promoters of the highest confidence for all genes were located within 400 bp upstream of coding sequences (data not shown). We therefore amplified a fragment of ~ 400 bp upstream of each gene and inserted it into the multiple cloning site (MCS) before the *E. coli lacZ* gene in pHGEI01. After integration and antibiotic marker removal, mid-log phase cultures (~0.3 of OD_600_) were prepared for the β-galactosidase assay. Cysteine-treated samples were collected 30 min after cysteine addition. As shown in Figure 2C, *lacZ* expression driven by the *cysM* and SO_1095 promoters fluctuated within a narrow range, indicating that impacts of the addition of cysteine on their expression were negligible. In contrast, expression levels of the *cysK*, *sseA*, *metB*, *metC*, and *mdeA* genes were significantly different in cultures between the normal and cysteine-induced conditions. Given that cysteine is the product of CysK, reduction in *cysK* expression is expected upon the addition of the amino acid. The remaining four genes showed increases in their expression levels to varying extent, depending on the gene. Consistent with the data presented in Figure 2A, the *sseA* and *mdeA* genes were expressed substantially higher in the treated samples, confirming that SseA and MdeA are cysteine-inducible enzymes involved in H_2_S generation in *S. oneidensis*. In the case of the *metB* and *metC* genes, the induction is not surprising because cysteine is a substrate of the pathway in which they play a role (Figure S1). Moreover, similar results were obtained from an independent examination of expression levels of these genes by using qRT-PCR (Figure S3). Taken together, these data conclude that MdeA is the predominant enzyme for H_2_S generation from cysteine and SseA makes a significant contribution when cysteine is abundant.

### SirACD and PsrABC are independent of each other

**Figure 3:**
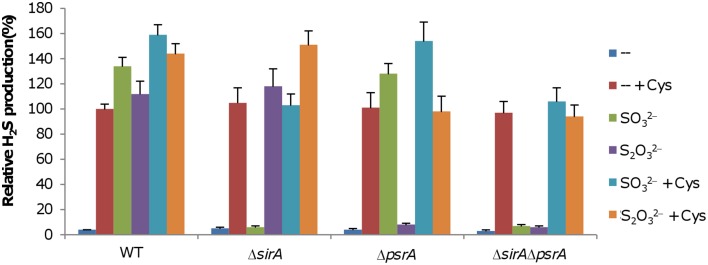
**Characteristics of H_2_S generation by *S. oneidensis* strains under anaerobic conditions**. Fumarate of 5 mM was used as EA to support growth to ~0.2 of OD_600_, which was then added with either SO^2−^_3_ or S_2_O^2−^_3_. H_2_S generation was measured 8 h after the addition. Averaged H_2_S level of the wild-type in the presence of cysteine was set to 100% for subsequent normalization as stated in Figure 2. “EA + Cys” represents the indicated EA plus cysteine. Data are presented as the mean ± SD from at least four independent experiments.

With SO^2−^_3_, S^0^, and S_2_O^2−^_3_ as the sole EAs, *S. oneidensis* is able to generate H_2_S by using the SirACD and PsrABC complexes, respectively (Burns and DiChristina, 2009; Shirodkar et al., 2011). However, whether these two complexes interplay with each other is worth studying given that many sulfur species are enzymatically convertible from one another (Kimura, 2014). To this end, we constructed Δ*sirA*, Δ*psrA*, and Δ*sirA*Δ*psrA* strains and compared their capacities of H_2_S generation to that of the wild-type. When grown on fumarate, H_2_S generation in the Δ*sirA*, Δ*psrA*, and Δ*sirA*Δ*psrA* strains was extremely low, resembling that in the wild-type (Figure 3), implicating that loss of SirACD, PsrABC, or both has no effect on the cysteine degradation pathway under the condition. In the presence of SO^2−^_3_, H_2_S levels in cultures of the wild-type and Δ*psrA* strains increased dramatically (Figure 3), an observation in excellent agreement with the results of a previous study (Shirodkar et al., 2011). Similarly, robust H_2_S generation was observed from the wild-type and Δ*sirA* strains with S_2_O^2−^_3_ as the sole EA. In both cases, the Δ*sirA*Δ*psrA* strain produced H_2_S at levels comparable to that of the wild-type grown on fumarate. In the presence of cysteine, H_2_S generation was further enhanced in the wild-type, suggesting an additive effect from respiration of sulfur species and cysteine degradation, which was also evident in Δ*sirA* with S_2_O^2−^_3_ and in Δ*psrA* with SO^2−^_3_. Expression of each of these genes *in trans* in its corresponding mutant restored H_2_S generation to the wild-type levels, confirming that the observed phenotypes were due to the intended mutations (Figure S4). These results indicate that the SirACD and PsrABC complexes are fully responsible for dissimilatory H_2_S generation, and appear functionally independent of each other.

### Crp is a global regulator essential for endogenous H_2_S generation

Given that many enzymes participate in H_2_S generation simultaneously, we became interested in whether there is a regulator mediating all of the processes in which these enzymes are involved, not necessarily in a direct manner. In our effort to identify such a regulator, we took advantage of transposon-based random mutagenesis. A random mutant library was generated using pHGT01, which allows screening for active operons as well as cryptic ones because of an embedded promoter within the transposable fragment (Fu et al., 2013; Yin et al., 2015). The medium was supplemented with proper amounts of cysteine, SO^2−^_3_ and S_2_O^2−^_3_, such that one of the significant contributors alone can produce a sufficient amount of H_2_S for being a H_2_S-plus strain. In approximately 10,000 gentamycin-resistant isolates screened, we found ~200 showing substantial reduction in H_2_S generation by using Pb(Ac)_2_-socked paper strips. However, many of them turned out to be false positive during subsequent validation. Among the remaining, two mutants were completely negative in H_2_S generation whereas three generated H_2_S at levels just detectable. Insertions in the former two were mapped into the *crp* gene and in the latter three were mapped into the *cyaC* gene. In *S. oneidensis*, Crp is the most important global regulator for both aerobic and anaerobic respiration and is activated by binding to cAMP, whose production mainly depends on membrane-bound adenylate cyclase CyaC (Saffarini et al., 2003; Charania et al., 2009). The insertions found in both *crp* and *cyaC* genes strongly suggest that the Crp regulatory system likely plays a crucial role in endogenous H_2_S generation in this microorganism.

**Figure 4:**
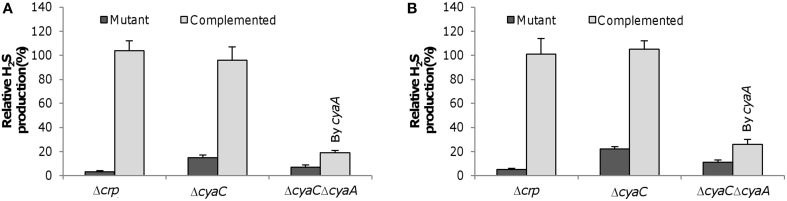
**Crp-cAMP is essential to H_2_S generation by *S. oneidensis*. (A)** H_2_S levels in anaerobic cultures of indicated mutants with SO^2−^_3_ and S_2_O^2−^_3_ at 2 mM each were measured and normalized to values of the wild-type. TMAO of 5 mM was used as EA to support growth to ~0.2 of OD_600_, which was then added with SO^2−^_3_ and S_2_O^2−^_3_. **(B)** H_2_S levels in aerobic cultures of indicated mutants with cysteine at 2 mM were measured and normalized to values from the wild-type. In both **(A,B)** The double mutant (Δ*cyaC*Δ*cyaA*) was complemented by the *cyaA* gene. Data are presented as the mean ± SD from at least five independent experiments.

To confirm this, we constructed a *cyaC* in-frame deletion strain (Δ*cyaC*) and examined the H_2_S generation capacity of the resulting strain, along with a *crp* deletion strain (Δ*crp*). The H_2_S generation assay revealed that the Δ*crp* strain completely lost the ability to produce H_2_S whereas the Δ*cyaC* strain retained residual capacity, approximately 17% relative to the wild-type when grown with sulfite and thiosulfate anaerobically (Figure 4A). This scenario can be readily explained by the presence of other adenylate cyclases, CyaA and CyaB, the former of which displays a detectable, albeit weak, ability to synthesize cAMP (Charania et al., 2009). Additional removal of the *cyaA* gene from the *cyaC*^−^ background further reduced H_2_S generation to a level comparable to that resulting from the *crp* deletion. Similar results were obtained with cultures grown with 2 mM cysteine under aerobic conditions (Figure 4B), manifesting that Crp is also required for H_2_S generation from cysteine degradation. The essentiality of Crp to H_2_S generation from both processes was further supported by similar findings observed from cultures grown with sulfur, thiosulfate, and 2 mM cysteine under anaerobic conditions (Figure S5). The observed phenotypes resulting from the *crp* or *cyaC* deletions were corrected by their expression *in trans*, confirming that the defect in H_2_S synthesis was due to the intended mutations (Figure 4).

### Crp directly controls transcription of psrA and sirA but not *mdeA*, SO_1095, or sseA

**Figure 5:**
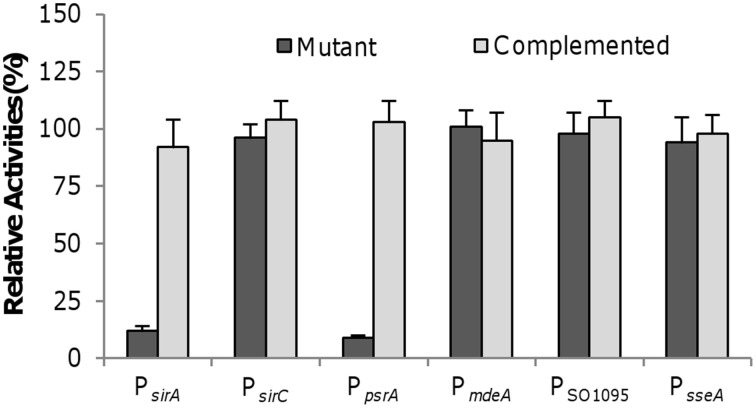
**Crp on activities of indicated promoters**. For each test gene, the promoter-*lacZ* construct was introduced into the chromosome of the Δ*crp* strain and the activities of the promoter as single copy were assessed by β-galactosidase assay. The same assay was performed with the complemented mutant for comparison. Data are presented as the mean ± SD from at least four independent experiments.

Given that Crp is the master regulator for anaerobic respiration and directly controls transcription of many genes encoding terminal reductases and their immediate regulators (Saffarini et al., 2003; Dong et al., 2012; Zhou et al., 2013), we predicted a similar scenario for the *sirACD* and *psrABC* genes, or at least some of them. The *psrABC* genes are predicted to be co-transcribed but the *sirA* and *sirCD* genes belong to two separate operons. To test whether these operons are under control of Crp, the promoters for these operons were predicted and fragments covering the promoter sequences were generated and placed in the front of the *E. coli lacZ* gene within pHGEI01 for β-galactosidase assay. In the Δ*crp* strain, P*_sirA_* and P*_psrA_* were not responsive to SO^2−^_3_ and S_2_O^2−^_3_, respectively (Figure 5). However, the loss of Crp did not significantly affect the activity of P*_sirCD_*. We then assessed the impact of the *crp* deletion on expression of the *mdeA*, SO_1095, and *sseA* genes. Surprisingly, none of these genes was negatively affected by the loss of Crp with respective to expression, even in the presence of cysteine (Figure 5).

In a previous study, operons under direct control of Crp were predicted by screening the *S. oneidensis* genome to identify those with conserved Crp-binding motifs in their upstream region (Gao et al., 2010b). The presence of such motifs in front of both the *sirA* and *psrABC* operons is in line with our expression data presented above. To confirm there is direct interaction between Crp and the upstream region of the *sirA* and *psrABC* operons, we performed bacterial one-hybrid (B1H) analysis. The B1H assay, a robust technique applicable to a wide variety of different transcriptional factor families, used here is derived from the BacterioMatch II two-hybrid system (Guo et al., 2009). To detect interaction, vectors containing “bait” (DNA) and “target” (DNA-binding regulator) are co-transformed into BacterioMatch II Validation Reporter Competent Cells, of which those having positive DNA–protein interactions are able to grow on 3-amino-1,2,4-triazole (3-AT). To prepare the bait, a ~300 bp fragment centered by the predicted Crp-binding motif for each test gene was cloned into pBXcmT, which was paired with pTRG carrying the *crp* gene for co-transformation. Positive interactions from P*_sirA_*/Crp and P*_psrA_*/Crp were detected and confirmed by growth on plates containing both 3-AT and streptomycin (12.5 mg ml^−1^), contrasting all other test genes that failed to produce any colonies in 40 h on the selective or confirmation plates (Table 2). In summary, these data manifest that Crp, likely in a direct manner, activates transcription of the *sirA* and *psrABC* operons but has little impact on the genes involved in cysteine degradation.

**Table 2:** **Bacterial one-hybrid (B1H) assay of Crp with various promoters**.

**DNA**	**Regulator**	**Colonies on nonselective plates^a^**	**Colonies on selective plates^b^**	**Confirmation^c^**	**Interaction**
/–	/–	197	0	–	No
/–	/Crp	163	0	–	No
/P*_cyd_*	/–	179	0	–	No
/P*_cyd_*	/Crp	174	169	168	Yes
/P*_16S_*	/Crp	202	1	0	No
/P*_sirA_*	/Crp	183	168	155	Yes
/P*_sirC_*	/Crp	212	2	0	No
/P*_phsA_*	/Crp	149	143	139	Yes
/P*_mdeA_*	/Crp	192	1	0	No
/P*_SO_1095_*	/Crp	205	0	–	No
/P*_sseA_*	/Crp	183	3	0	No

a *M9 agar + 25 μg/ml chloramphenicol + 12.5 μg/ml tetracycline*.

b *a + 5 mM 3-AT*.

c *b + 12.5 μg/ml streptomycin*.

### ArcA directly represses transcription of the *mdeA* gene

**Figure 6:**
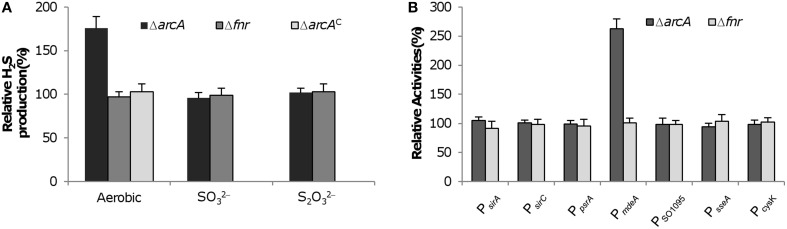
**Impact of loss of on H_2_S generation in *S. oneidensis*. (A)** ArcA and Fnr on H_2_S generation. H_2_S levels in cultures of indicated mutants grown aerobically or anaerobically with 10 mM SO^2−^_3_ or S_2_O^2−^_3_ as the sole EA were measured and normalized to values from the wild-type. Complementation of the Δ*arcA* mutant (Δ*arcA*^C^) was conducted with aerobic growth. **(B)** ArcA and Fnr on activities of indicated promoters. For each test gene, the promoter-*lacZ* construct was introduced into the chromosome of the Δ*crp* strain and the activities of the promoter as single copy were assessed by β-galactosidase assay. Data are presented as the mean ± SD from at least four independent experiments.

In *S. oneidensis*, the function of major global regulators, Crp, Arc, and Fnr, is substantially altered compared to their respective *E. coli* counterparts (Gao et al., 2010b). In *E. coli*, Fnr and Arc two-component system (TCS) are primarily responsible for the switch between aerobic and anaerobic metabolism and Crp is the decisive regulator in the process of carbon repression (Green and Paget, 2004; Deutscher et al., 2006). Interestingly, the *S. oneidensis* counterpart of *E. coli* Fnr has no significant role in these processes and Arc appears to be important in aerobic respiration and plays a limited role in anaerobiosis (Gralnick et al., 2005; Gao et al., 2008, 2010b), whereas Crp becomes the most important regulator mediating aerobic and anaerobic respiration (Saffarini et al., 2003; Dong et al., 2012; Zhou et al., 2013). Given the functional switches among these regulators, we intended to examine whether Fnr or Arc influences H_2_S synthesis in *S. oneidensis*. Under aerobic and anaerobic conditions, loss of Fnr did not show any noticeable effect on H_2_S generation, consistent with its overall dispensable role in respiration (Figure 6A). However, Arc, although negligible for H_2_S generation from anaerobic respiration, apparently was involved in regulation of cysteine degradation. In line with that Arc functions as a repressor in general, the loss of the system resulted in overproduction of H_2_S (Figure 6A).

To determine which genes are under influence of Arc, we measured the activity of promoters for all genes involved in H_2_S generation (Figure 6B). Compared to the wild-type, the strain missing the *fnr* gene was indistinguishable in expression of all test genes, supporting that Fnr has no role in regulation of H_2_S generation. In the case of Arc, although expression levels of the *sirA*, *sirCD*, *psrABC*, SO_1095, and *sseA* operons were barely altered in the *arcA* deletion strain, the *mdeA* gene was expressed significantly higher compared to the wild-type. This repression by Arc is probably a result of direct binding to the upstream region of the operon because it contains an ArcA-binding motif (Gao et al., 2008; Wang et al., 2008). Moreover, we assessed the impact of Arc on expression of the *cysK* gene as its loss leads to a H_2_S overproduction phenotype, similar to that resulting from the loss of ArcA. However, expression levels of the *cysK* gene were comparable between the *arcA* positive and negative strains (Figure 6B), eliminating a possibility that Arc plays a role in cysteine biosynthesis. These results conclude that Crp is essential for H_2_S generation and Arc specifically regulates transcription of the *mdeA* gene whereas Fnr is dispensable in *S. oneidensis*.

## Discussion

The purpose of this study was to identify predominant sources of H_2_S inside *S. oneidensis*. Previous studies on anaerobic respiration of sulfur-containing substances had demonstrated that PsrABC and SirACD enzyme complexes could catalyze H_2_S generation, provided that the corresponding EA was present (Burns and DiChristina, 2009; Shirodkar et al., 2011). PsrC and SirD belong to the NrfD/PsrC family of integral membrane proteins, which exclusively function as menaquinone oxidase/reductase (Simon and Kern, 2008). We provided evidence that these two complexes are predominantly, if not exclusively, accountable for H_2_S generation via respiration of sulfur species. Interestingly, there is no interplay between the complexes detected in this study, indicating that they do not release intermediates for each other during respiration.

In parallel, the genome of *S. oneidensis* encodes many homologs of enzymes in cysteine degradation, whose counterparts are accountable for H_2_S generation in mammals (Wang, 2012; Shatalin et al., 2011). By a combination of chemical and biological analyses, we determined that the predominant route for H_2_S generation is through the MdeA-based cysteine degradation. As a methionine γ-lyase, MdeA is highly homologous to *P. aeruginosa* CSE, able to use L-cysteine as a substrate, together with H_2_O, to produce pyruvate, NH_3_, and H_2_S (Sato and Nozaki, 2009). Additionally, we found that another CSE homolog and a 3MST homolog, SO_1095 and SseA respectively, contributed to H_2_S generation in *S. oneidensis*. Under aerobic growth conditions, MdeA accounts for at least two-thirds of H_2_S that is generated endogenously, regardless of the presence of cysteine. SO_1095, which is surprisingly not subjected to cysteine induction, catalyzes the reaction that releases H_2_S of ~20% in the absence of additional cysteine. On the contrary, SseA is highly inducible by cysteine but without the inducer its contribution is negligible. *S. oneidensis* probably does not possess functional CBS as CBS homologs appear to be cysteine synthases, suggesting that the bacterium, similar to those studied before such as *E. coli*, lacks at least one of enzymes for H_2_S generation via cysteine degradation (Shatalin et al., 2011).

In *E. coli*, the transcriptional regulator Fnr and the Arc TCS together control switches between aerobic and anaerobic respiration whereas Crp dictates carbon metabolism (Green and Paget, 2004; Deutscher et al., 2006). While Fnr of *S. oneidensis* has been reported to have a limited role in respiration of certain EAs (Maier and Myers, 2001; Cruz-Garcia et al., 2011), the data documented here, together with those presented previously, support the notion that the physiological significance of the regulator is marginal (Gao et al., 2010b; Fu et al., 2013; Zhou et al., 2013). The *S. oneidensis* Arc system is atypical, consisting of three components, ArcS, HptA and ArcA (Gralnick et al., 2005; Lassak et al., 2010; Shroff et al., 2010). Moreover, the regulons of the *E. coli* and *S. oneidensis* Arc systems have few components in common, suggesting a major difference in their physiological roles (Gao et al., 2008; Wang et al., 2008; Yuan et al., 2012). Several lines of evidence manifest that this three-component system functions primarily under aerobic conditions although it is required for dimethyl sulfoxide respiration, a phenomenon presumably arising from adventitious appearance of an ArcA-binding motif in front of the operon (Gao et al., 2008; Wang et al., 2008; Dong et al., 2014).

Instead, *S. oneidensis* employs Crp to control expression of most of respiratory reductases and, in some cases, their immediate regulators (Saffarini et al., 2003; Dong et al., 2012). Therefore, it is conceivable that expression of the *psrA* and *sirA* operons is mediated by Crp. By B1H analysis, we detected positive interactions between Crp and the upstream sequences of these two operons, manifesting that the regulation is carried out by Crp directly. Notably, the regulator of *S. oneidensis* resembles its *E. coli* counterpart in cAMP-dependent activation despite the substantial difference in Crp regulons between these two organisms (Charania et al., 2009; Zhou et al., 2013). This gains further support from the finding that loss of CyaC, the major cAMP synthetase, results in a phenotype similar to that caused by the Crp removal as shown here and before (Charania et al., 2009).

One of the most striking findings in this study is that Crp is also absolutely essential for H_2_S generation via cysteine degradation in *S. oneidensis*. We do not know yet the mechanism underlying this essentiality. Expression of the *mdeA*, SO_1095, and *sseA* genes is not affected by the Crp removal, indicating that Crp influences biological processes beyond these proteins. Given that the loss of Crp abolishes cysteine induction, we speculate that the regulator may play an essential role in controlling intracellular cysteine levels. Unlike Crp, the involvement of the Arc system in H_2_S generation via cysteine degradation is clear. The TCS represses expression of the *mdeA* gene likely in a direct control manner. To date, little is known about the environmental cues for *S. oneidensis* ArcS, but it is clearly different from the *E. coli* paradigm as the atypical system functions under both aerobic and anaerobic conditions (Gralnick et al., 2005; Gao et al., 2008; Yuan et al., 2012). We are working to decipher the mechanism by which Crp dictates H_2_S generation via cysteine degradation and to identify the signal triggering the Arc system.

### Conflict of interest statement

The authors declare that the research was conducted in the absence of any commercial or financial relationships that could be construed as a potential conflict of interest.
