# Supplementary material for: Endogenous generation of hydrogen sulfide and its regulation in Shewanella oneidensis
Source: Front Microbiol. 2015 Apr 28;6:374. doi: 10.3389/fmicb.2015.00374 (PMC4412017; doi:10.3389/fmicb.2015.00374)
Supplement: Supplementary file 1 [file Presentation1.PDF]

Supplemental materials of

Endogenous generation of hydrogen sulfide and its regulation in *Shewanella oneidensis*

Genfu Wu, Ning Li, Yinting Mao, Guangqi Zhou, and Haichun Gao*

Institute of Microbiology and College of Life Sciences, Zhejiang University, Hangzhou, Zhejiang, 310058, China

A

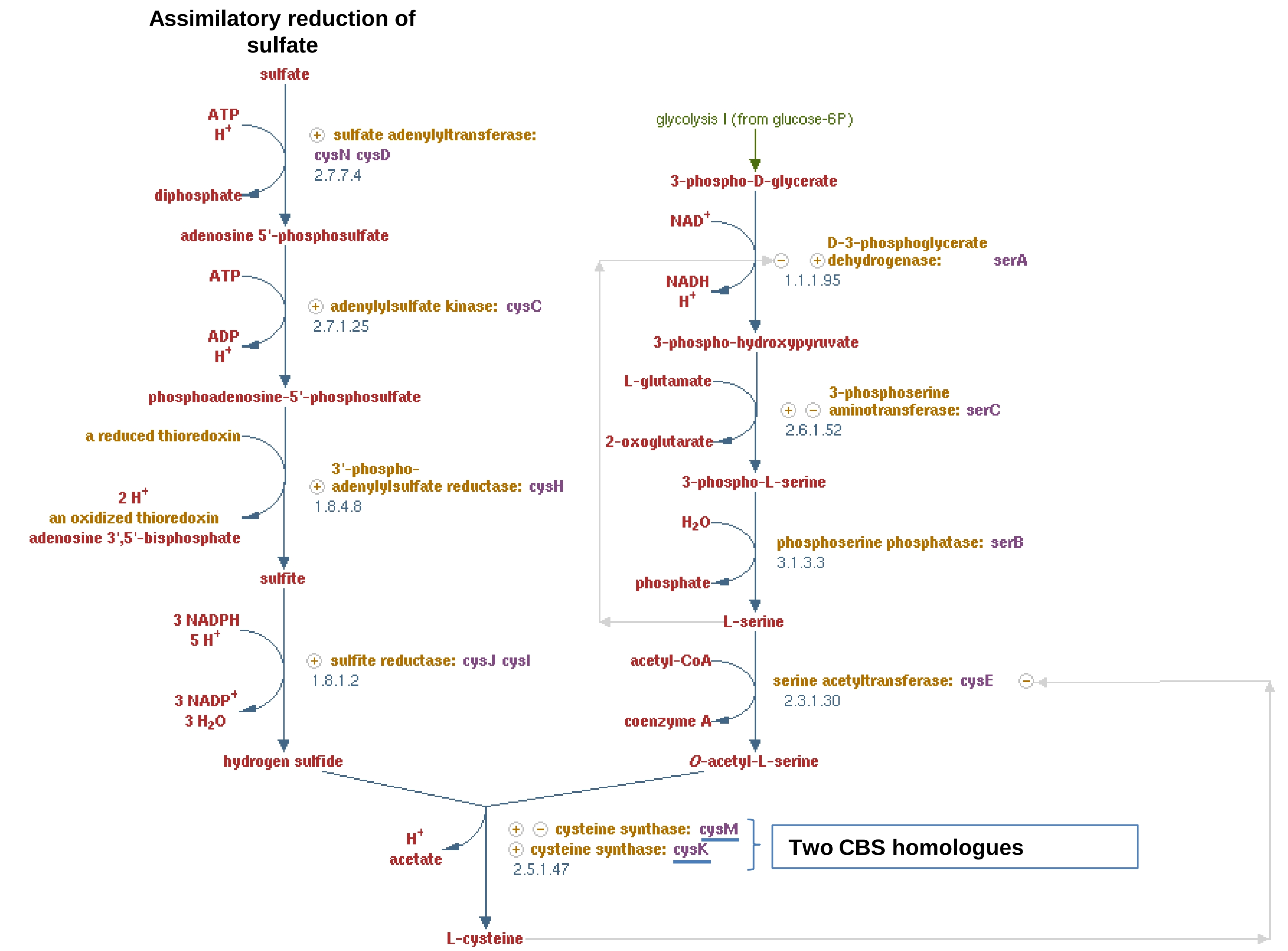

FIGURE S1 Enzymes proposed to be involved in endogenous generation of H_2S under aerobic conditions in *S. oneidensis*. The diagram is obtained from ecocyc.org and modified by adding relevant information. (Keseler et al., 2013). (A) Assimilatory reduction of sulfate and synthesis of L-cysteine. Note that two *S. oneidensis* CBS homologues catalyze the synthesis of L-cysteine from O-acetyl-L-serine and H_2S.

B

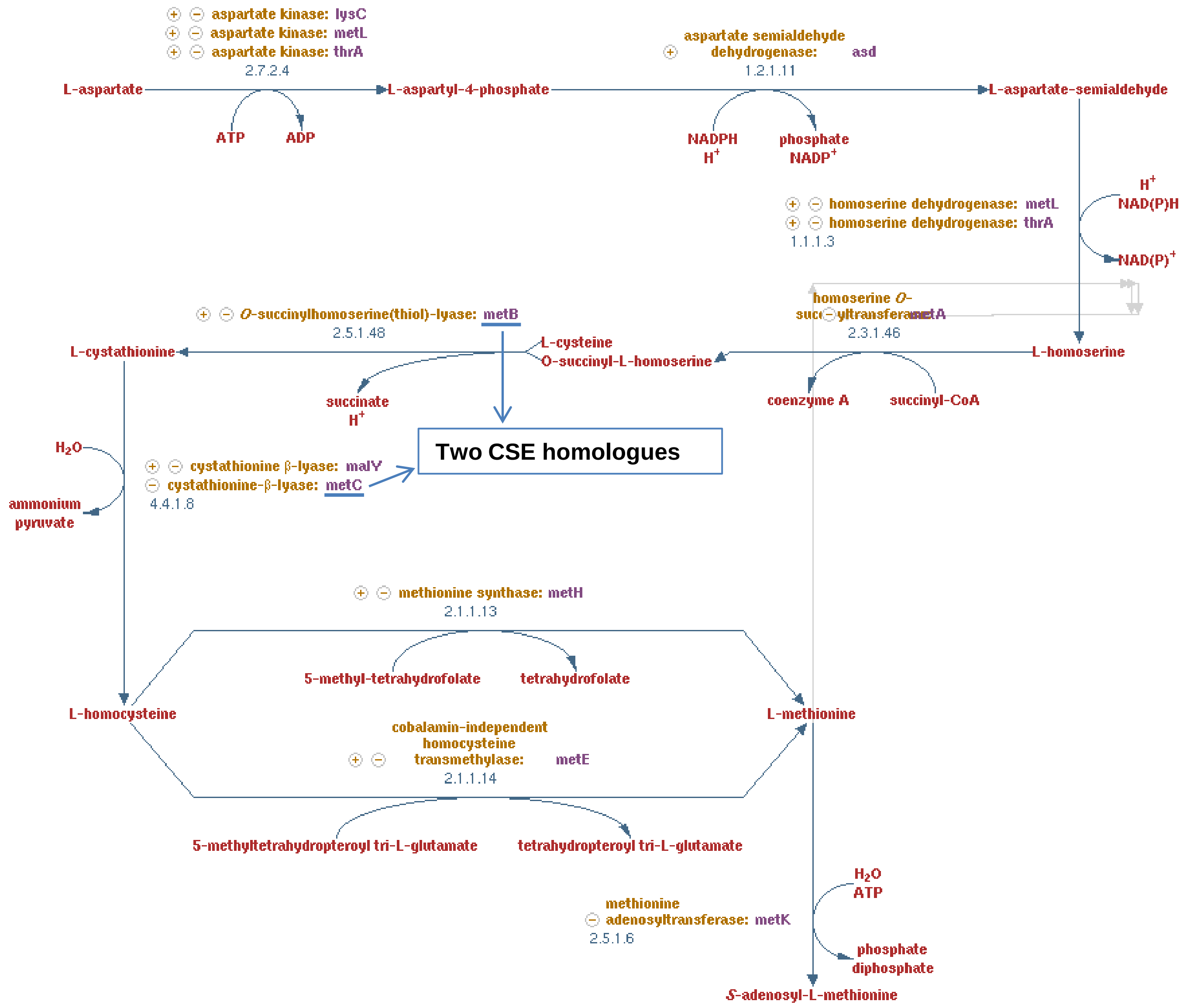

FIGURE S1 (B) Superpathway of *S*-adenosyl-L-methionine biosynthesis. Note that two *S. oneidensis* CSE homologues, MetB and MetC, likely funciton in the pathway.

C

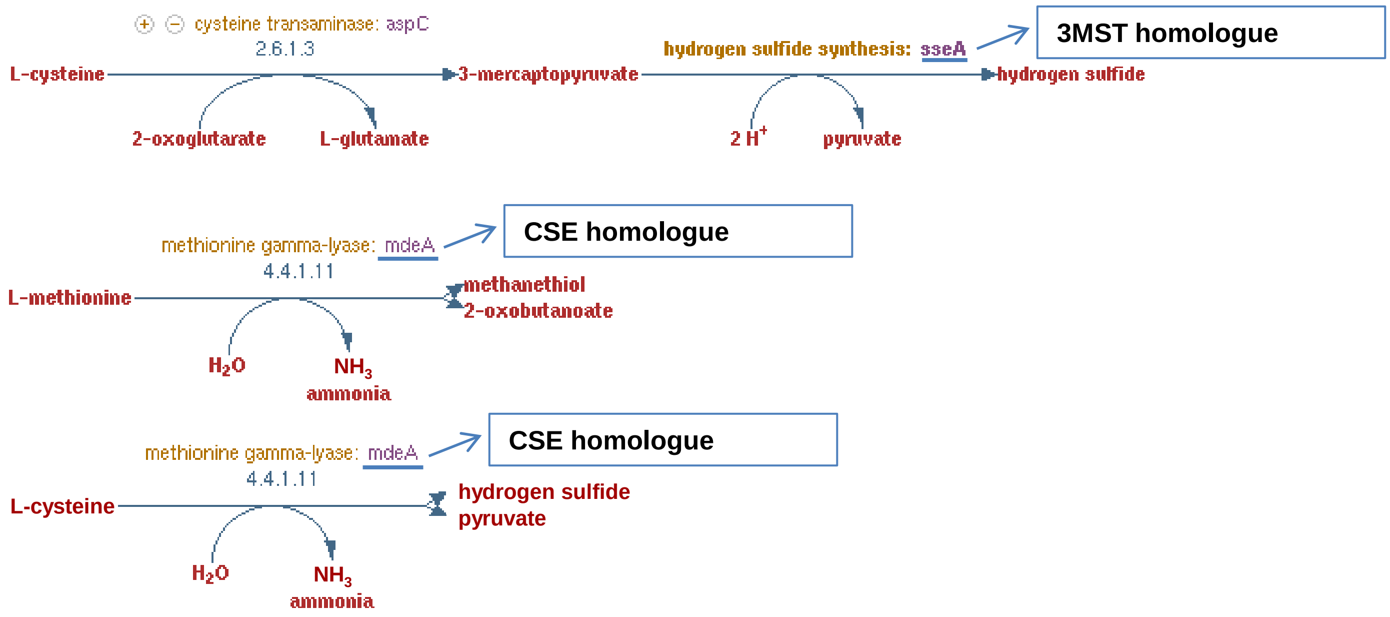

FIGURE S1 (C) Catalytic reactions of SseA (upper) and MdeA. MdeA, a *S. oneidensis* CSE homologue, can use both L-methionine (middle) and L-cysteine (lower) as substrates but produce H_2S only from L-cysteine.

A.

```
SO1261          MEYP-LVSTQWLEEQLTSPELVLLDASMAVVLGKEPILYHEPIC--IPRSRRFDVEEDFC 57
EcSseA          MSTTWFVGADWLAEHIDDPEIQIIDARMASPGQEDRNVAQEYLNGHIPGAVFFDIEA-LS 59
                *. . :*.::** *:: .**: ::** **    ::  : :* :   ** :  **:*  :.

SO1261          DKTSTQIHALPRFESFVEGIAKLGIEPQSLIVIYDNQGIYSSPRAWWIFKVMGFHRVYVL 117
EcSseA          DHTSPLPHMLPRPETFAVAMRELGVNQDKHLIVYDEGNLFSAPRAWWMLRTFGVEKVSIL 119
                *:**.  * *** *:*. .: :**:: :. :::**: .::*:*****:::.:*..:* :*

SO1261          DGGLPQWIAEDRVTSSRYQEEGIDYGVTDSAPLAAVLQYQSAKVMDAEAVLARLDDSESA 177
EcSseA          GGGLAGWQRDDLL---------LEEGAVELPEGEFNAAFNPEAVVKVTDVLLASHENTAQ 170
                .***. *  :* :          :: *..: .       ::.  *:..  **   .:. :

SO1261          IIDARGATRFLGQVSEPRPGVRSGHIPHSVNLPFGEVLNGYKMKSTTELQAIFQAL-VGN 236
EcSseA          IIDARPAARFNAEVDEPRPGLRRGHIPGALNVPWTELVREGELKTTDELDAIFFGRGVSY 230
                ***** *:** .:*.*****:* **** ::*:*: *::.  ::*:* **:*** .  *.

SO1261          KALRIFSCGSGITACILILASVVAGHKSAVLYDGSWADWGSRTDLPIER-- 285
EcSseA          DKPIIVSCGSGVTAAVVLLALATLDVPNVKLYDGAWSEWGARADLPVEPVK 281
                .   *.*****:**.:::** .. .  .. ****:*::**:*:***:*
```

B.

```
SO3598          -----MTIEACIGQTPLVRLQRLDCGSSTVLLKLEGNNPAGSVKDRAALNMINQAELRQE 55
SO2903          MSKIFEDNSYTIGNTPLVRLNRVSQG--KVLAKVESRNPSFSVKCRIGANMIWDAEKKGL 58
Pa-Cbs          --------LDLIGNTPLVRVTRFDTGPCTLYLKLESQNPGGSIKDRIGVAMIEAAERDGR 52
                           **:*****: *.. *   .:   *:*..**. *:* *  .   **  **

SO3598          IAPGDTLIEATSGNTGIALAMAAAIKGYKMILIMPSNSTQERKDAMQAYGAELLLVD--- 112
SO2903          LTKDHELIEPTSGNTGIALAYVAAARGYKLTLTMPNTMSLERRKLLKALGANLVLTEGAK 118
Pa-Cbs          LRPGGTIVEATAGNTGLGLALVGRAKGYRVVLVVPDKMSTEKVLHLRAMGAEVHITRSDV 112
                :  .  ::*.*:****:.** ..  :**:: * :*.. : *:   ::* **:: :.

SO3598          ---NMEAARDLALALQAEGKGK--VLDQFNNQDNANAHFLTTGPEIWQQSQGKITHFVSS 167
SO2903          ---GMKGAIDKAEEIRQSAPEKYILLQQFNNPANPEIHEKTTGPEIWNDTDGAVDVFVAG 175
Pa-Cbs          GKGHPEYYQDVAARLAQDIPGAF-FADQFNNPANPLAHECGTGPELWAQTGHDLDAIVVG 171
                      :   * *  :  .       . :****  *.  *   ****:* ::   :  :* .

SO3598          MGTTGTIMGVSKYLKSRN-PDITIVGLQPADG----SSIPGIRRWP---------QEYLP 213
SO2903          VGTGGTITGVSRYLKKVAGKAITSVAVEPADSPVITQTLAGLPVQPGPHKIQGIGAGFIP 235
Pa-Cbs          VGSSGTLTGLTRFFQKVQ-PELEMVLADPEGSIMAEYSRSGTLGTPGSWAVEGIGEDFVP 230
                :*: **: *::::::.     :  *  :* ..      : .*    *         ::*

SO3598          GIFDAARVDLMMDIEEQDAKAMARALAREEGICAGVSSGGAVYAALELAR--QYPGSVVV 271
SO2903          GNLDIELIDRVETVTNDEAIEMARRLMQEEGILVGISSGAAVVAANRIAALPEFADKTIV 295
Pa-cbs          AIADLSSVRHAYSISDEESFAMARELLRVEGIPGGSSTGTLLAAALRFCR-EQKEPKRVV 289
                .  *   :     : ::::  *** * : ***  * *:*  : ** .:.   :   . :*

SO3598          AIVCDRGDRYLSSGLFS---------- 288
SO2903          VVLPSAAERYLSSVLFQGQFGDEENIQ 322
Pa-Cbs          SFVCDTGTRYLS-KIYNDQWMTDQG-- 313
                 .: . . ****  ::.
```

C.

```
Pa-Cse          MSQHDQHPDAPAQAFATRVIHAGQAPDPSTGAIMPPIYANSTYIQESPG---------VH 51
SO4056          MTEGKLVTER---QLATLAVRQGIESDTQYGAVVPPIYLSTNYAFDGHK---------NP 48
SO1812          ----MQDKSSKMWKAATQAIHAGHEREAFG-SLVTPLYQTATFVFDSAQQGGERFA-GNE 54
SO1095          ------------MKLESLALHHGYESEATTKAAAVPIYQTTSYTFDDTQHGADLFD-LKV 47
SO2191          --------MTDKHQLATQIVSVGRDKKWTKGVINPPVFRASTIVFDTMEDMRHAAKNKTN 52
                :  :  *   .        *::  :.   :

Pa-Cse          KGLDYGRSHNPTRWALERCVADLEGGTQAFAFASGLAAIS-SVLELLDAGSHIVSGNDLY 110
SO4056          REFDYSRSGNPTRSILGDALAKLEKGATGVVTCTGMAAIT-LVTTLLGPDDLLVVPHDCY 107
SO1812          PGYIYTRLGNPTVAELERKMAILERAEAAAATASGMGAVSAALLANLQIGDHLVASNAVY 114
SO1095          AGNIYTRIMNPTTSVLEQRLAAIEGGIGALAVASGMAAITYAIQALTQVGDNIVSTSQLY 107
SO2191          GEMFYGRRGTPTHFAFQAAVSELEGGAGTALYPSGAAAISAALLSFLQAGDHLLMVDSVY 112
                    * *  .**   :   :: :* .       :* .*::  :      .. ::     *

Pa-Cse          GGTFRLFERVRRRSAGHRFSFVDPTDLQAFEAALTPETRMVWVETPSNPLLRLTDLRAIA 170
SO4056          GGSYRLFTNLAKKGQ-FKLLVVDQTDNQALAQAIAQQPKMVWIETPSNPLLRVVDIEAIA 166
SO1812          GCTFALMTNQFARFG-IEVTLVDFSDVELIERAIKPNTKVIFCETPVNPHLQVFDLSAIA 173
SO1095          GGTYNLFAHTLPRQG-VEVRMAAFDDFEELEALIDAKTKALFCESIGNPAGNIVDLKRLA 166
SO2191          EPTRDFCSHILAGFN-IETTYYDPLIGEGIRALIRPNTKVLFLESPGSITMEVQDVPTLC 171
                  :  :  .        .         : :   :  :.: :: *:  .   .: *:  :.

Pa-Cse          QLCRARGIISVADNTFASPYIQRPLELGFDVVVHSTTKYLNGHSDVIGGIAIVGDN---- 226
SO4056          KASHGVGALVVVDNTFLSPILQQPLLLGADIVIHSTTKYINGHSDVVGGAVIAKD----- 221
SO1812          AIAKRHSLTSIVDNTFMTPLLQQPIALGIDVVVHSATKYLNGHGDVIAGIVCGS------ 227
SO1095          EIAHKHGVPLIVDNTVATPVLCRPFEHGADIVIHSLTKYIGGHGTTIGGIIIDSGKFDWV 226
SO2191          RIAHEHGLVTILDNTWASPINSKPFEMGVDVSIQAATKYIVGHSDVMIGTATAN------ 225
                  .:  .   : ***  :*   :*:  * *: ::: ***: **. .: *

Pa-Cse          ----------------------------PDLRERLGFLQNSVGAISGPFDAFLTLRGVKT 258
SO4056          ----------------------------PQIGETLHWWSNTLGLTGSAFDSYQTLRGLRT 253
SO1812          ---------------------------EAQLHRVKYEILKDIGAVMSPHDAWLILRGLKT 260
SO1095          ANKERFSLLNQADPSYHGVVYTEAFGPAAFIGRCRVVPLRNTGAALSPHSAFLLLQGLET 286
SO2191          ----------------------------EQYWPQLRERSYLLGQTTSPDDVYLATRGLRT 257
                                          *   .. . :   :*:.*

Pa-Cse          LALRMERHCSNALALAQWLERQPQVARVYYPGLASHPQHELAKRQMRG--FGGMISLDLR 316
SO4056          LAVRIREHQRNAQRIVELLSNSPVVSKVYYPGLADHPGHAIAAKQQKG--FGAMLSFELK 311
SO1812          LDVRLQRHCESAQRVAEFLEQHPAVTRVYYPGLKSHSGHRFIGQQMR--RAGGVIAFELA 318
SO1095          LSLRMERHCANALALAEYLILHPSVSWVNYGALPSSP-FRENCEKITGGKASGIISFGIK 345
SO2191          LGVRMAQHEKNALKVANWLQTRPEVDHLRHPAFETCPGHEFFKRDFSA--SNGLFSFVLK 315
                * :*: .*  .*  :.: *   * *  : : .:   . .    ..      ..:::: :

Pa-Cse          CDLAGARRFLE-----NVRIFSLAESLGGVESLIEHPAIMTHASIPAETRADLGIGDSLI 371
SO4056          GGEAEVVAFLD-----ALSLFSVAESLGGVESLVAVPATMTHRAMEPQARFEAGIKDTLL 366
SO1812          ADFTQAMAFVG-----NLKLFSIAVSLGDAESLIQHPASMTHSPYSPEARAAAGIGDNLL 373
SO1095          AATPEEGKIAGGKFIDALKMVLRLVNIGDAKSLACHPASTTHRQLDANELARAGVSEDLI 405
SO2191          QGDQEAVTALVEN----MQHFKMGFSWGGYESLILG----IFGIERIRSATKWDASKPLI 367
                                 :  .    . *. :**        .     .     .  . *:

Pa-Cse          RLSVGVEALEDLQADLAQALAKI--------- 394
SO4056          RLSVGIEDADDLVADIQAGLAAVAACQ----- 393
SO1812          RISVGLEDCDDIIADLSQALA-ALV------- 397
SO1095          RISVGIEHIDDIIADVSQALEKALV------- 430
SO2191          RVHIGLEDPEDLIADLSAGFERFNAVLAAKVK 399
                *: :*:*  :*: **:  .:
```

FIGURE S2 Sample sequence alignments of H_2S-producing enzymes in *S. oneidensis*. (A) 3MST from *E. coli* (EcSseA) and its *S. oneidnesis* ortholog. (B) and (C) CBS and CSE from *P. aeruginose* (Pa-Cbs and Pa-Cse) and their *S. oneidnesis* orthologs. Conserved amino acids are highlighted. Active-site loop residues of HS 3MST and CBS are shown in red. Residues in bold indicate key active-site residues of CSE.

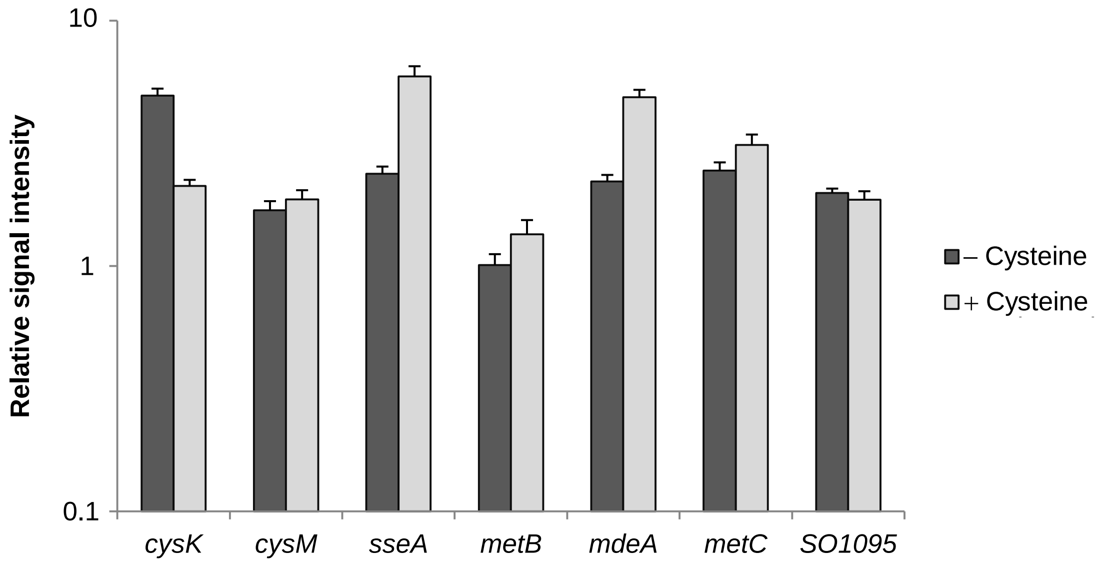

FIGURE S3 Impact of cysteine on expression of various genes. qRT-PCR analysis of RNA extracted from mid-log growing cells. All data were normalized to expression of the 16s rRNA gene, which was constant during the exponential growth phase. Numbers reported are standardized to expression of the reference gene. Error bars represent standard deviation for triplicate cultures.

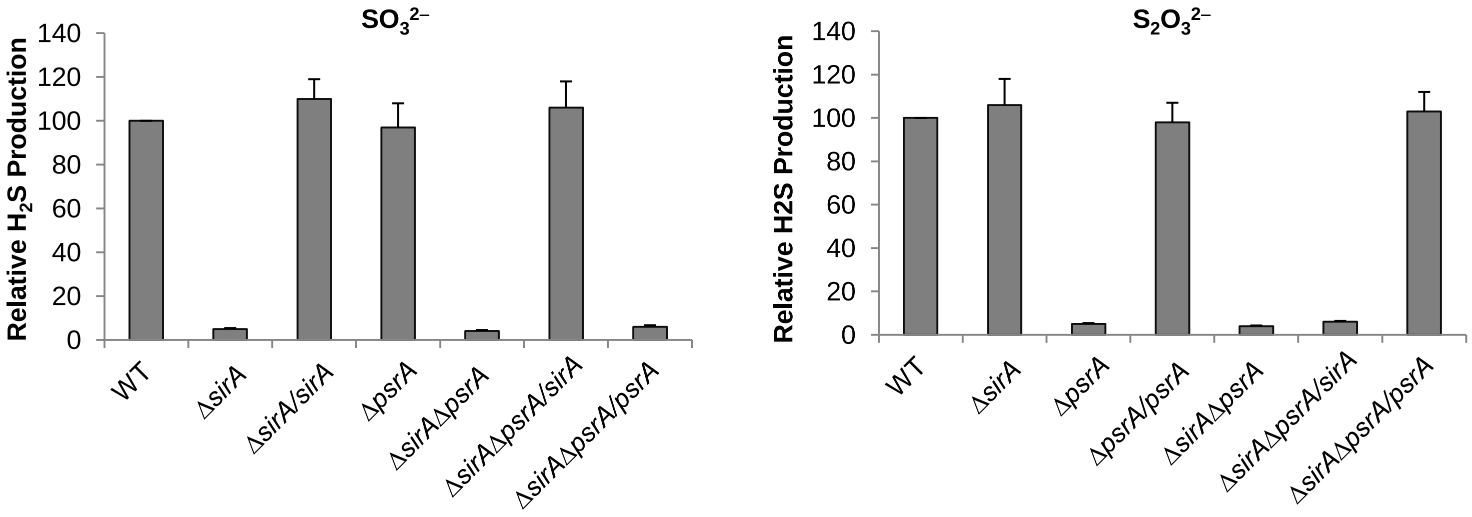

FIGURE S4 Complementation of Δ*sirA*, Δ*psrA*, and Δ*sirA*Δ*psrA* with respect to H_2S generation under anaerobic conditions. Fumarate of 5 mM was used as EA to support growth to ~0.2 of OD_{600}, which was then added with either SO_3^{2-} or $S_2O_3^{2-}$. H_2S generation was measured 8 hours after the addition. Averaged H_2S level of the wild-type with each EA was set to 100% for subsequent normalization. Data are presented as the mean ± SD from at least 4 independent experiments.

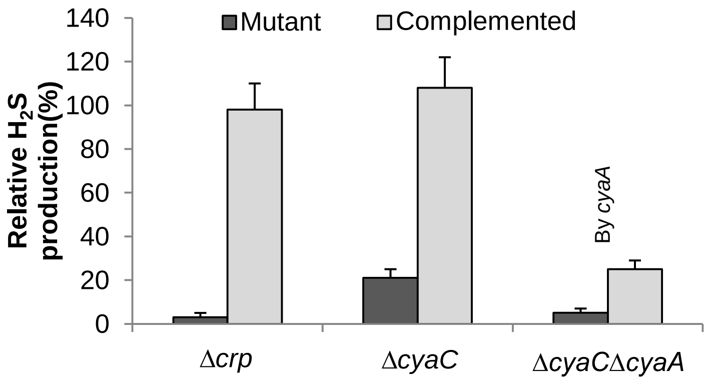

FIGURE S5 Crp-cAMP is essential to H_2S generation by *S. oneidensis*. Cells were cultivated as described in FIGURE4. H_2S levels in cultures of indicated mutants with SO_3^{2-}, $S_2O_3^{2-}$, and cysteine at 2 mM each were measured and normalized to values of the wild-type. The double mutant (Δ*cyaC*Δ*cyaA*) was complemented by the *cyaA* gene. Data are presented as the mean ± SD from at least 5 independent experiments.

TABLE S1 Comparison of $3MST_{Ec}$, CBS_{Pa} and CSE_{Pa} and *S. oneidensis* proteins[a]

Locus	Gene	Predicted Function	$3MST_{Ec}$	CBS_{Pa}	CSE_{Pa}
SO1261	*sseA*[b]	mercaptopyruvate sulfurtransferase	56%/9e^{-50}		
SO3598	*cysM*	cysteine synthase B		55%/3e^{-50}	
SO2903	*cysK*	cysteine synthase A		54%/9e^{-49}	
SO4056	*metB*	cystathionine γ-synthase			63%/3e^{-102}
SO1812	*mdeA*	methionine γ-lyase			62%/1e^{-95}
SO1095		O-acetylhomoserine (thiol)-lyase			50%/2e^{-54}
SO2191	*metC*	cystathionine β-lyase			51%/4e^{-51}

[a]Both sequence similarities and E-values were given.

[b]Named in this study
